# Remote learning among students with and without reading difficulties during the initial stages of the COVID-19 pandemic

**DOI:** 10.1007/s10639-021-10559-3

**Published:** 2021-04-24

**Authors:** Joanna Zawadka, Aneta Miękisz, Iwona Nowakowska, Joanna Plewko, Magdalena Kochańska, Ewa Haman

**Affiliations:** 1 Faculty of Polish Studies, University of Warsaw, Krakowskie Przedmieście 26/28, 00-927 Warsaw, Poland; 2 Faculty of Psychology, University of Warsaw, Warsaw, Poland; 3 Institute of Psychology, The Maria Grzegorzewska University, Warsaw, Poland; 4 Laboratory of Language Neurobiology, Polish Academy of Sciences, Nencki Institute of Experimental Biology, Warsaw, Poland; 5 Educational Research Institute, Warsaw, Poland

**Keywords:** COVID-19, Dyslexia, Reading, Remote teaching, Remote learning, Higher education

## Abstract

This article presents the results of a survey on yet under-researched aspects of remote learning and learning difficulties in higher education during the initial stage (March – June 2020) of the COVID-19 pandemic. A total of 2182 students from University of Warsaw in Poland completed a two-part questionnaire regarding academic achievements in the academic year 2019/2020, living conditions and stress related to learning and pandemic, as well as basic demographic information, and *Dyslexia Diagnosis Questionnaire* (DDQ). The analyses were carried out in three sub-groups of students: who self-reported having a formal diagnosis of dyslexia (CDYS), self-reported reading difficulties, but had no formal diagnosis of dyslexia (SIDYS), and who reported no reading difficulties (CON). The results of the survey revealed that compared with the CON group, more students from CDYS and SIDYS groups did not pass at least one exam in the summer semester. CDYS and SIDYS groups experienced higher stress due to epidemiological restrictions, they had more difficulties than CON with the organisation of learning and obtaining credit during the COVID-19 pandemic. The results indicate a need for special consideration of additional support for students experiencing reading difficulties (whether or not they have a formal diagnosis).

## Introduction

The outbreak of the COVID-19 in March 2020 caused universities around the world to close the campuses and libraries, limit face-to-face contacts and switch to remote teaching in a very short time (Crawford et al., 2020; Zhao et al., 2020). Poland was no exception. This sudden need to rely only on web-based teaching can be looked at as an unexpected radical change in the way we approach learning and teaching. Although the new technologies allowing online learning already existed however, the majority of courses offered by universities were face-to-face courses and it was not that popular to incorporate new teaching/learning solutions offered by e-learning platforms (Zhao et al., 2020). Online learning evolved from distance learning (Moore et al., 2011) which was popularized in Europe by the Open University, established in the UK already in 1969 (Woodley, 1981; Zhao et al., 2020). There are an apparent diversity and lack of consensus on the terminology used to refer to learning over the internet. Distance learning, online learning, remote learning have been used interchangeably (see e.g. Chigeza & Halbert, 2014; Ali, 2020; Carrillo & Flores, 2020). We decided to use the term remote learning as opposed to the term online learning as there are opinions (e.g. Hodges et al., 2020 propose even a term “emergency remote teaching”) that there is a need to distinguish between online teaching understood as planned in advance to remote teaching/learning which was implemented during the pandemic and was rather a spontaneous substitute of face to face approach.

Many educational institutions conducted online surveys to evaluate the level of satisfaction of remote learning, the effectiveness of remote teaching, and to learn how students were coping with stress related to the COVID-19 pandemic (Aristovnik et al., 2020; Demuyakor, 2020). The results generally indicate several factors playing an important role in efficient remote studying: availability of a good quality Internet infrastructure and digital equipment, teaching staff support, computer skills in using remote learning platforms and programs, living conditions, as well as motivation, self-discipline, and self-initiative (Aristovnik et al., 2020; Daniels et al., 2021; Zhu et al., 2020). The effectiveness and the ease of implementation of remote learning depended much on the prevalence of this form of learning before the COVID-19 pandemic (Aristovnik et al., 2020).

Although since the beginning of the COVID-19 pandemic, all students were undoubtedly affected by sudden and radical changes to all areas of their life, the group of students with developmental learning disabilities and other impairments deserves special consideration.

In their study, Forteza-Forteza et al. (2021) explored the learning processes of children/youth with dyslexia (aged 9–21, mostly from primary and secondary educational level) during the COVID-19 pandemic and the school lockdown. The authors argue that most pupils in their sample had access to technology, but only a small percentage had used applications that aided their reading, writing, and text comprehension. Despite the number of studies addressing the problems of students with reading difficulties (or more generally – students with learning disabilities) in the higher education context, only a small part of it refers to e-learning, distance learning, online learning etc. in students with dyslexia (Cataudella et al., 2021). To the best of our knowledge, to date no study has explored learning difficulties of any kind experienced by higher education students with dyslexia in remote learning during the COVID-19 pandemic. The aforementioned article of Forteza-Forteza et al. (2021) does not refer to individuals at the university level. And yet this group is potentially vulnerable to experience educational drawbacks due to the current situation. We aim to fill the research gap by looking at the situation of students with reading difficulties during the shift to remote learning due to the COVID-19 pandemic. There is a need to explore the situation of this group in order to prepare guidelines for developing university support and to implement changes in the educational process, if necessary.

### Literature review

Dyslexia is a specific learning disability characterized by difficulties with accurate and fluent word recognition, poor decoding abilities, and/or poor spelling (WHO, 2015). In consequence, a person with dyslexia may have problems with reading comprehension, which in turn may lead to a reduced reading experience, limited growth of vocabulary and general knowledge (IDA, 2002). It has been documented that the gap in reading between typical readers and dyslexic readers persisted (despite an improvement) from early school years to adolescence (Ferrer et al., 2015; Shaywitz et al., 1999; Snowling et al., 2007) and adulthood (Reis et al., 2020; Snowling et al., 2020). Reis and colleagues (Reis et al., 2020) reviewed 178 studies of adults with dyslexia and concluded that compared to matched control groups the adults with dyslexia performed poorly on all of the writing and reading tasks except for reading comprehension. The authors point out the need for studies devoted to dyslexic adults as they experience life-long symptoms which are still far from being understood. Mortimore and Crozier (2006) have reported results of a survey administered among 136 students attending 17 higher UK education institutions. Note-taking, organisation of essays, and expressing ideas in writing were reported as academic skills that dyslexic students found difficult. It was also noted that although the difficulties had different patterns, they were experienced by those students also at earlier levels of education. Moreover, individuals with dyslexia may require more family support and professional help throughout their educational careers (Moojen et al., 2020) while facing not only difficulties in learning. Secondary symptoms such as high anxiety and low self-esteem are often present in children (Alexander-Passe, 2008) and maybe maintained in adults. Research results indicate a higher anxiety level in university students who were diagnosed with dyslexia compared to students without learning difficulties (Carroll & Iles, 2006; Nelson et al., 2015; for contrary findings see: Lamm & Epstein, 1992), lower self-esteem (Riddick et al., 1999). Moreover, dyslexia is often accompanied by other disorders. Rates of comorbidity between specific reading difficulties and other neurodevelopmental disorders varied in the range between 11 and 70% (Moll et al., 2014, for an overview). Co-occurring disorders can modulate the severity of dyslexic difficulties over time and additionally modulate the symptoms of learning disabilities in the dyslexic population.

Symptoms of dyslexia in adulthood may not be as severe as in childhood, but they still make an impact on a person's daily life and educational career achievements (Hellendoorn & Ruijssenaars, 2000) that in turn is associated with worse perspectives of having stable, high skilled employment, and ultimate educational attainment (Maughan et al., 2020; Rutter & Yule, 1975). Adults with dyslexia may present slower and more labour-intensive reading and writing (Beidas et al., 2013; Moojen et al., 2020; Suárez-Coalla & Cuetos, 2015; Tops et al., 2013). Students with dyslexia may have difficulties in coordinating the sequence of actions when performing activities (Fawcett & Nicolson, 2008; Stoodley & Stein, 2013), difficulties in automating activities (Fawcett & Nicolson, 1992), deficits in implicit learning (Menghini et al., 2006), difficulties with short term memory (Tamboer et al., 2017), and working memory (Jeffries & Everatt, 2004). Due to the above-mentioned deficits, people with dyslexia may have problems with the organisation of their work and they need much more time to read and complete written tasks (Reid, 2016). For them, reading may not only be a tedious and tiring activity in itself, but they also may need to read the text many times to understand it (Reid, 2016). In their qualitative research Woodfine and colleagues (Woodfine et al., 2008) suggested that students with dyslexia experience a lack of confidence and encounter problems when engaged in text-based synchronous learning activities related to reading, spelling, sentence structure, transposition, memory, organisation, and time management. Taking this into account, one may expect that students with dyslexia will encounter greater difficulties than non-dyslexic students when learning online. Other research investigated the effects of different computer-based media in learning materials on the learning outcomes of individuals with and without dyslexia and pointed to possible advantages. The results of a study by Taylor et al. (2007) showed that animated learning materials have the potential to help students with and without dyslexia. According to the respondents, animated learning materials facilitate understanding concepts more than text or pictures. Beacham and Alty (2006) pointed out that different dyslexic students may benefit from using e-learning materials if the media used to present information corresponding to a particular preferred learning style. However, those benefits refer to particular materials and do not consider the complexity of the new approach to teaching and learning. It can be expected that even after the COVID-19 pandemic, hybrid/blended learning, which is a combination of online and classroom face-to-face instruction (Kaur, 2013; Chigeza & Halbert, 2014) becomes a norm in the academic world. Therefore a holistic approach to its possible outcomes should be taken.

An unexpected implementation of remote teaching due to pandemic can be seen as a challenge and a chance to reveal which aspects of the new approach to teaching need to be further explored in order to assure equal learning opportunities for all the students.

Based on the literature related to the difficulties experienced by adults with dyslexia, and the exploratory nature of our study we propose the following hypotheses:

- (H1) Dyslexic (CDYS) students struggle more than non-dyslexic students (CON, SIDYS) with effects of the pandemic (higher level of stress, lower academic achievement, more frequent reports of difficulties with remote learning).
- (H2) Group of students with self-diagnosed dyslexia (SIDYS) has more difficulties with remote learning than the control group (CON), but less than the group with formal diagnosis (CDYS).

### The COVID-19 epidemic in Poland and its influence on higher education

In Poland, the first confirmed case of COVID-19 was recorded on the 4th of March 2020 (Pinkas et al., 2020). As early as seven days later all Polish universities began suspending stationary classes and in the next few weeks they gradually transitioned to remote learning (Sidor & Rzymski, 2020). On the 20th of March 2020 a state of epidemic emergency was declared (Pinkas et al., 2020). The strict restrictions of national quarantine lasted till the 20th of April 2020. In that time many lockdown-type control measures were introduced, among others: use of public spaces remained banned, citizens were encouraged to strict self-isolation and social distancing, social life and physical activities were reduced to the minimum, non-family gatherings were limited to two people, non-essential travel was forbidden, individuals were required to wear face coverings in public spaces and keep a social distance of minimum 1,5 m, minors were forbidden go outside unaccompanied. The state of epidemic emergency introduced in March–April 2020 influenced negatively the psychological well-being of Polish university students raising the level of depression, anxiety, and stress (Debowska et al., 2020; Rogowska et al., 2020; Szczepańska & Pietrzyka, 2021; Talarowska et al., 2020; Wieczorek et al., 2021).

The functioning of higher education institutions was limited starting from the 12th of March until the 25th of March 2020. Since all universities were obliged to conduct solely remote learning, Rectors of many Polish universities issued the ordinance, which suspended the learning process until the 25th of March when the implementation of information and communication technologies (ICT) for the remote teaching process was to be accomplished. Although Moodle learning platforms had been used prior to the pandemic (Maleńczyk & Gładysz, 2019; Półjanowicz et al., 2013; Rosak-Szyrocka & Wojciechowski, 2015; Suchanska & Keczkowska, 2007; Szadziewska & Kujawski, 2017) not all academic teachers had experience in working with it neither with Google Suite for Education, Skype, Zoom nor with Microsoft Teams, etc. since the majority of curricula were realised in a stationary manner. Due to unexpected and rapid changes in the teaching and learning process many universities decided to conduct online surveys during the semester and after it, which were to investigate the perception of remote learning. The results showed that remote learning during the initial stage of the pandemic was considered less effective than face-to-face learning in terms of increasing skills and social competencies compared to traditional classes, students assessed their active participation during online classes to be lower (Bączek et al., 2021).

### Current study

The general aim of our study was to explore the effects of the COVID-19 pandemic on the quality of learning, living conditions, academic performance, and stress levels of university students with special consideration of students with reading difficulties.

In the current paper, we particularly analysed how students who experience reading difficulties cope with remote learning during the pandemic. We compared three groups of students: (1) students with a formal diagnosis of dyslexia (confirmed dyslexia, CDYS), (2) students who reported dyslexic difficulties but did not have an official diagnosis (self-diagnosed dyslexic symptoms, SIDYS), and (3) a group of students which did not report reading problems (control, CON).

## Method

### Participants and procedure

The study was performed as a self-report anonymous survey conducted online between the 16th and 31st of July 2020 in one of the largest universities in Poland – University of Warsaw. The study was financed by an external grant, which aims at the improvement of existing and development of innovative solutions to increase the accessibility of education for students with disabilities. Thus, the study had the practical purpose of exploring the needs of a particular group of students. The strength of such an approach is that the policy of the university and ways of remote learning were the same for all of the participants. The conditions under which they learnt were thus similar, but at the same time, due to the variety of disciplines taught in this institution, the sample was diverse in terms of the fields of study such as science and humanities. The sampling was thus purposive (Teddlie & Yu, 2007) and steps were taken to reach as large a number of students of the particular university as possible. Students were recruited through a central administration mailing list, encompassing all students of the university was used to disseminate the link, and social media (fan pages of faculties, student groups) as well as snowball approach. Social media were used as support in advertising the information about the survey. The study was prepared on Qualtrics XM. There was no time limit, it was possible to return to previous questions, as well as to save the answers and finish the survey later. The participants were not compensated for their participation. They were asked to generate an individual code that can be used to match participants' responses if they participate in scheduled follow-up studies.

All participants were of an average age of 22.55 years. Out of 3510 students who took part in the study, 2182 participants completed the whole set of questionnaires. Power analysis conducted in G*Power 3.1 (Faul et al., 2007, 2009) indicated that this sample size would allow for the detection of a large effect (0.4) in ANOVA analysis (alpha = 0.05) with a power of 1.00. Only 4 participants declared having a COVID-19 diagnosis but 465 (21%) reported having self-diagnosed COVID-19 symptoms, 79 students were officially in quarantine. Majority of the participants (1290, 59%) were students of humanities, economics (300, 14%), formal sciences (232, 11%), biology/medical (67, 3%) and 16% (293) were students from other, interdisciplinary fields. First-year students made up 38% (823) of the whole group, second-year – 32% (676), third – 17% (378), forth – 7% (148) and fifth – 5% (113). Additionally, 26% of the respondents changed their place of living after the beginning of the COVID-19 pandemic.

To identify students with possible reading problems, participants were asked whether they had dyslexia (formal diagnosis, self-diagnosis due to experienced reading difficulties, no diagnosis). 216 students declared having formal diagnosis (CDYS) and 201 reported reading difficulties but no formal diagnosis (SIDYS). A control group of students without reading difficulties (CON) was selected. To maximize the accuracy and power of the study, we matched participants belonging to the CON group to those belonging to the CDYS group by the use of the optimization algorithm (Kuhn, 1955) implemented via MATLAB (The Math-Works Inc. Natick, MA, USA). Table 1 presents general information describing the sample.

**Table 1:** General descriptive statistics of the sample

General information	All	CON	CDYS	SIDYS
Number of participants	2182	216	201	216
Mean age	22.55	22.23	22.27	22.53
Gender (female/male)	1609/517	133/72	133/72	138/53
COVID-19 positive (tested)	4	0	0	1
Job loss	353	28	33	39
COVID-19 symptoms in family members	156	18	20	14
Changed place of residence	575	54	43	52
Passed all exams in summer semester (PE)	1555	41	71	68
Did not pass all exams in summer semester (NPE)	557	167	136	125

The participants were also asked whether they suffer from any neurodevelopmental disorder, behavioural disorder, or affective disorder. 60 participants (27.8%) in the CDYS group, 47 students in the SIDYS group (23.4%), and 11 participants (5.1%) in the CON group declared having co-occurring disorders.

### Measures

We used a two-part questionnaire. The first one aimed to collect basic demographic information, as well as information related to the issues concerning the sudden change from traditional to remote learning due to the COVID-19 pandemic such as increased stress due to the COVID-19 pandemic and due to remote learning, changes in work and living conditions, advantages and disadvantages of remote studying in the summer semester, and academic achievement (measured by success in passing the exams).

In the analysis presented below we mainly focus on students' answers to the following statements:

- *Dyslexia diagnosis. *We asked students “Do you have a diagnosis of dyslexia?” and answers were: (1) Yes, (2) I do not have a formal diagnosis of dyslexia but I have such difficulties, (3) I do not have a formal diagnosis of dyslexia and I have no such difficulties.
- *Exam success.* We asked students if they passed all the exams during the winter and summer semesters.
- *Stress due to epidemiological restrictions.* We asked students to score on a scale ranging from 1 to 100, where 1 – nothing changed, 100 – a drastic increase, how much the epidemiological restrictions (implemented by the Polish government in March 2020) changed their level of general stress. A similar scale ranging from 1–100 was used by Zajenkowski and colleagues (Zajenkowski et al., 2020) to measure COVID-19-related phenomena.
- *Stress due to the forms of remote learning.* We asked students to score the stress related to (1) different forms of passing subjects; (2) weekly homework; (3) the need to adapt to remote learning; from -2 (not stressful at all) to 2 (very stressful).
- *Amount of work needed to get credit*. We asked students how much work they have to put into getting credit for the subjects in both semesters. They answered on a scale from 1 (very little) to 5 (a lot).
- *General difficulties due to remote learning.* We asked students how they perceived difficulties related to (1) remote learning, such as adjusting the pace and time of work to their own needs; (2) contact with the lecturers; (3) general aspects of remote learning. They answered on a scale from 1 (I strongly disagree) to 5 (I fully agree with this statement) (the choice of items is supported by similar studies e.g. Abbasi et al., 2020; Adnan & Anwar, 2020; Serhan, 2020).

For the full text of all the questions, see Table 2.

**Table 2:** Full list of questions, statistics and direction of observed differences

Question	CON/SIDYS/CDYS comparison	
	Statistics	Direction of differences
Score your stress related to:		
Weekly homework	*F*(2,567) = 6.308, *p* < 0.01	CON < CDYS, SIDYS
Presentations on the forum of the group in the video-conference mode	*F*(2,269) = 7.476, *p* = 0.001	CON < CDYS, SIDYS
Tests with short open answers	n.s	–
Papers, essays written with a time limit to get credit for the course	n.s	–
Tests with closed questions on the e-learning platform	n.s	–
Tests on the e-learning platform with a set time limit	n.s	–
Tests with the inability to return to the previously given answer during the test	n.s	–
Tests with penalty for the wrong answer	n.s	–
Oral credits in the video-conference mode	*F*(2,446) = 5.129, *p* < 0.05	CON < CDYS < SIDYS
The need to learn to use new IT tools by participating in remote learning (Kampus platform, Google tools and/or others)	*F*(2,603) = 3.776, *p* < 0.05	CON < CDYS, SIDYS
The need to acquire knowledge about the equipment and technologies needed for the time or exam (camera, microphone, computer, high-speed Internet)	*F*(2,620) = 9.864, *p* < 0.001	CON < CDYS, SIDYS
How much do you agree with these statements:		
I am able to complete the tasks on time	*F*(2,630) = 6.792, *p* = 0.001	CON > CDYS, SIDYS
I feel more under time pressure on remote deadlines than I did when the classes were held in class	*F*(2,630) = 14.019, *p* < 0.001	SID > CDYS > CON
E-learning requires more writing and reading than classroom teaching	*F*(2,630) = 3.129, *p* < 0.05	SIDYS > CON
It is more difficult for me to contact a lecturer than when teaching was done in the classroom	*F*(2,630) = 6.918, *p* = 0.001	CDYS, SIDYS > CON
It is more difficult for me to complete subjects in remote learning than in class	*F*(2,630) = 8.618, *p* < 0.001	CDYS, SIDYS > CON
During remote learning, I can better adjust my learning pace to learning—for example, I can take more breaks and work at different times of the day	n.s	-
During remote learning, I can better adapt the learning to my schedule—I can play audio or video recording every time I need it	*F*(2,630) = 3.225, *p* < 0.05	CON > SIDYS
During remote learning, I am more motivated to learn because I control what and when I learn	n.s	-
It is hard for me to study remotely because I'm unfamiliar with new technologies	*F*(2,630) = 4.922, *p* < 0.01	CDYS, SIDYS > CON
It is more difficult for me to study remotely, because it requires programs, which I did not use at the university before	*F*(2,630) = 3.367, *p* < 0.05	SIDYS > CON
It is more difficult for me to understand a remote lecture than a classroom one	n.s	–
It's harder for me to work on a computer because there are more distractions (social networks, e-mail, news sites)	n.s	–
It is easier for me to study remotely because I have the impression that the teachers treat the student more individually	n.s	–
It is harder for me to study remotely because I feel anonymous	n.s	–
It is easier for me to study remotely, all the necessary aids are in place and I do not have to spend time looking for them in the library	n.s	–
I feel that I am more involved in studying remotely than in in-site mode	n.s	–
If you were having difficulties during remote learning, could you have referred to the lecturer and/or support provider?	n.s	–

*n.s* non-significant

Additionally, in order to estimate the magnitude and presence of self-reported reading difficulties, Dyslexia Diagnosis Questionnaire (DDQ, original: Kwestionariusz Diagnozy Dysleksji, Bogdanowicz, 2012) was used. DDQ is a short, self-report questionnaire inspired by Adult Dyslexia Checklist (Smythe & Everatt, 2001) and Vinegrad’s Revised Adult Dyslexia Checklist (Vinegrad, 1994). Vinegrad’s Revised Adult Dyslexia Checklist is a widely used, standardized questionnaire enabling to differentiate participants in terms of the intensity of reading difficulties (Snowling et al., 2012). The DDQ questionnaire consists of 30 items, e.g. *Do you read slower than other people?; Do you have difficulties speaking, especially in stressful situations, for instance publicly?; Do you have difficulties in understanding the instructions that you read?* The participants answer on a 4-point Likert scale ranging from 1 (definitely not) to 4 (definitely yes). In the current study, the alpha reliability for DDQ was Cronbach’s alpha = 0.92 (standardized = 0.92).

### Statistical analysis

All exploratory statistical analyses were conducted in SPSS 26.0 Statistics software for Macintosh. All the analyses were conducted on three groups based on dyslexia’ diagnosis. The analysis of descriptive statistics was conducted to illustrate the demographic and other selected characteristics of the students responses.

For numeric variables, chi-square test was used and for continuous variables parametric tests for means (ANOVA with S–N-K post hoc comparison). The significance level was determined at p < 0.05.

### Ethics

The study materials and design have been approved by the University of Warsaw research ethics committee. The survey was conducted following the 1964 Declaration of Helsinki with later amendments. All participants provided informed consent and were informed about the possibility of quitting the survey anytime without consequences. The survey was distributed by the administrative staff of the University. The survey was not conditional on any type of student’s evaluation connected to passing any classes and the format of the questionnaire did not allow to identify the student personal data nor learning outcomes.

## Results

**Fig. 1:**
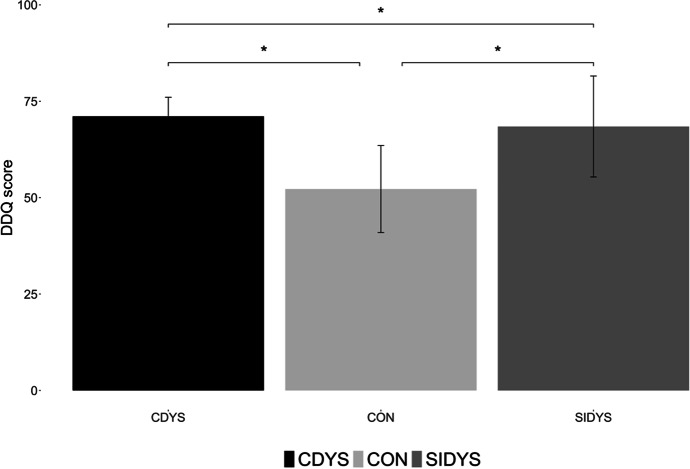
The average DDQ score in groups of students who declared diagnosed dyslexia (CDYS), declared self-diagnosed dyslexia (SIDYS), and without dyslexia (CON)

Analysis comparing the average score in symptoms of reading difficulties measured by DDQ (minimum = 30, maximum = 120) for three groups indicate that all the groups differ, *F*(2,630) = 123.98, *p* < 0.001, with S-N-K post-hoc comparison, alpha = 0.05. CON having the lowest score and SIDYS the highest (Fig. 1).

### Stress due to epidemiological restrictions

**Fig. 2:**
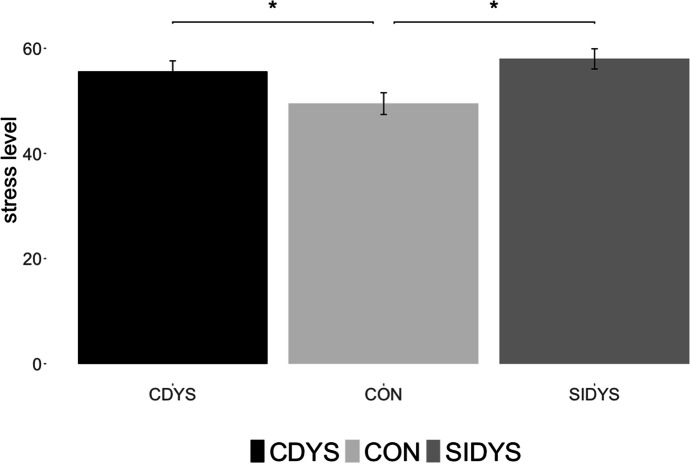
Groups average stress levels related to the epidemiological restrictions due to the COVID-19 pandemic

CON students reported lower levels of stress-related to the epidemiological restrictions implemented by the government than CDYS and SIDYS groups (*F*(2,610) = 4.616, *p* = 0.01; post-hoc S-N-K alpha = 0.05; see Fig. 2). The results support H1.

### Stress due to the forms of remote learning

Students’ assessment of the level of stress related to different forms of getting course credit ranging from −2 (not stressful at all) to 2 (very stressful) differ for weekly homework (*F*(2,567) = 6.308, *p* < 0.01; post-hoc S-N-K, alpha = 0.05; CDYS, SIDYS > CON) as well as for the need to adapt to remote learning (*F*(2,599) = 7.915, *p* < 0.001; post-hoc S-N-K, alpha = 0.05; CDYS, SIDYS > CON). The results support H1.

See Table 2 for the full list of additional questions and statistics.

### Exam success

The comparison of the number of the students who did not pass all of the exams in the three groups (CDYS, SIDYS, CON) revealed that compared with the CON group more students from CDYS, χ^2^ = 19.64, *p* < 0.001, and SIDYS, χ^2^ = 21.96, *p* < 0.001, groups did not pass at least one exam in the remote, summer semester. The results support H1 and H2.

### Perceived difference between winter and summer semester in the amount of work needed to get credit

Paired *t*-test analysis confirmed that for all three groups, the summer semester (March – June 2020) required more work and commitment than the previous winter semester (September 2019 – January 2020), both for completing the classes, CDYS: *t*(191) = 2.070, *p* = 0.04; CON: *t*(195) = 4.154, *p* < 0.001; SIDYS: *t*(186) = 3.65, *p* < 0.001, and workshops, CDYS: *t*(193) = 2.111, *p* = 0.036; SIDYS: *t*(185) = 2.45, *p* = 0.015. The results support partially H1 and H2.

### Difficulties in organisation of learning during the COVID-19 pandemic

Students also differed in terms of perceived facilitations and limitations related to remote learning, such as adjusting the pace and time of work to their own needs, ease of contact with the lecturers, and other general aspects of remote learning. The main pattern of differences was that CDYS and SIDYS groups had more difficulties than CON. The results partially support H1 and H2. See Table 2 for the list of questions and detailed statistics.

## Discussion

The COVID-19 pandemic has changed numerous areas of social life, including university education. Although studies on coping and the mental state of young adults during the pandemic in Poland are available (Debowska et al., 2020; Gambin et al., 2021; Nowakowska, 2020; Szczepańska & Pietrzyka, 2021), our study was pioneering in terms of focus on students with reading difficulties, who might be differently affected by the pandemic and remote learning due to the character of their difficulties in reading and writing. The issue of computer-assisted learning and online learning in dyslexic students was elaborated previously but in different (non-pandemic) contexts (Pang & Jen, 2018; Perelmutter et al., 2017; Woodfine et al., 2008). Students with dyslexia are present in a fair number at the university level and rising (López-Escribano et al., 2018; Mortimore & Crozier, 2006; Pino & Mortari, 2014) and thus understanding their situation during the remote learning period is worthwhile to support inclusion in university settings (for a review of the matter of inclusion of students with dyslexia in higher education see Pino & Mortari, 2014). In the current study, we explored how they faced the new situation and what were their main challenges during the time of rapid change in the teaching and learning process due to the COVID-19 pandemic. The results of our study partially confirm the hypotheses that (H1) dyslexic students struggle more than non-dyslexic students with effects of the pandemic (higher level of stress, worse academic achievement, more frequent reports of difficulties with remote learning) and (H2) which stated that a group of students with self-diagnosed dyslexia (SIDYS) has more difficulties with remote learning than the control group (CON), but less than the group with formal diagnosis (CDYS). The current study has shown how students who self-reported having a formal diagnosis of dyslexia or reading difficulties without a formal diagnosis and controls, differed in terms of their perception of (1) level of stress (regarding epidemiological restrictions and regarding changes in the forms of learning), (2) the difference in the perceived amount of work needed to get credit, and (3) the general difficulties due to remote learning.

The comparisons of answers in three groups of students (CDYS, SIDYS and CON), revealed significant differences. Our results indicate that there is indeed a need for looking into the issue of additional support for university students with reading difficulties due to changes in teaching introduced during the pandemic. Even before the pandemic period, it was indicated that such support is vital to enhance the functioning of dyslexic students at university (MacCullagh et al., 2017). During the pandemic, all students had to face new challenges, however the students from the control group (without reading problems) less often failed exams, had a lower level of perceived stress due to epidemiological restrictions and due to the forms of remote learning. The control group also reported fewer problems with remote learning. All groups (DYS, SIDYS, CON) claimed that the workload during the online semester was greater than during the in-site winter semester which can be due to the specificity of remote learning, lecturers' substantive and technical competencies related to the remote teaching as well as students’ technical competencies. As a result of sudden changes in the first months of the COVID-19 pandemic, many academic lecturers in Poland could provide only (or mainly) asynchronous remote teaching in which students were given tasks for self-study based on written materials (Cicha et al., 2021). This form of teaching requires relatively more reading and writing, which may be one of the reasons underlying the intergroup differences observed. Also, later changes to more synchronous teaching (using videoconferencing) aimed at increasing students' active participation in remote learning by encouraging them to explore problems by themselves, collect needed information beforehand, and present the results of the research. At this point, the question arises whether the increased workload in participating and completing classes translates to higher effectiveness of learning and better learning outcomes? This however goes beyond the scope of the current analysis.

We would like to draw attention to the similarities of reported difficulties in groups with a formal diagnosis and those without, but reporting reading problems. In the case of some of the answers, the CDYS and SIDYS groups did not differ significantly, but they differed from the assessments presented by the CON group. In other cases, assessments of the respondents from SIDYS ranked even higher than CDYS. Both the discrepancy and equality of the assessments highlight the need for supporting students experiencing dyslexia-like difficulties who may have not received formal assistance and support due to the lack of documented history of learning difficulties. It further indicates that reading problems constitute a real issue that should be taken into account when we think about appropriate student services (Pirttimaa et al., 2015; Quick, 2013; Reis et al., 2020). Individuals without a formal diagnosis of dyslexia may have not obtained it at the elementary-school level because of a mild severity of their problems and the failure to meet all diagnostic criteria. The formal diagnosis of dyslexia may be a reason for seeking reading comprehension therapy, which may be beneficial to the compensating strategies for dyslexic problems (Cancer et al., 2020; Galuschka et al., 2014, 2020; Pape-Neumann et al., 2015). People who did not have a formal diagnosis but feel they experience difficulties might be therefore disadvantaged by the fact that they did not receive treatment in the past. Our findings advocate for inviting adult students without diagnoses, but reporting difficulties in learning to undergo an assessment of their strengths and weaknesses in academic functioning to receive appropriate support in the higher education context. We hope that our findings encourage exploration of possibilities for appropriate support for students with dyslexia as apparently students with reading difficulties also struggle more with learning during the pandemic. Further studies are needed in order to disentangle the effects of new approaches to teaching and those due to pandemic.

### Limitations

Our work has several limitations that need to be taken into account when interpreting the results of the study. Firstly, the participants were students of one of the largest and most renowned universities in Poland therefore their reading skills might have been generally high. However, if we were able to obtain such differences in this population of highly functioning students, it only supports the need for further investigation of these issues in different educational settings. It should also be noted that the study was cross-sectional, making it impossible to form any causality statements and limiting the scope of understanding how people coped with learning before the COVID-19 pandemic. Moreover, the study based on self-report of reading problems, which might have made it difficult for some individuals to disclose the problems they encounter in real life, even though the study was anonymous and independent from academic assessment (there is a similar concern about their report of comorbid disorders). Stress-related variables were also measured with single items, which might have limited the scope of understanding the nuances of stress experienced by the students. In the interpretation of the results related to the problems with technical aspects of the remote learning, we did not control the level of technological skills prior to COVID-19 pandemic. We need to rely on knowledge that we have about the general situation in Poland and at the investigated university. Poland seems to be similar to other countries in terms of e.g. availability of resources to learn how to use digital devices and in some aspects (e.g. computer access) even exceeded averages across OECD countries (Organization for Economic Co-Operation and Development). As for the University of Warsaw since the establishment in 1999 of The Centre for Open and Multimedia Education (currently Digital Competence Center) new technologies are present and available to University of Warsaw community, including e-learning and online teaching. This unit provided very broad support and training long before the COVID-19 pandemic. However, the online courses were only supporting the on-site classes, whereas the pandemic moved all interactions and all courses to a remote mode.

### Future research directions

We postulate to continue research on the impact of the COVID-19 pandemic on learning and teaching at the universities, by the use of standardized questionnaires, as well as surveys tailored to the circumstances in which the students function in a given higher education institution. The question is whether the disproportions in problems experienced by students with and without reading difficulties will be observable even after the COVID-19 pandemic, when the on-site classes will be returned (even to some extent) or when the COVID-19 threat will be lowered (e.g., after vaccinating the population of students). It is also interesting whether now disadvantaged groups like students with dyslexia (or more broadly saying—with specific learning difficulties) would be further disadvantaged due the remote learning as the pandemic continue or will they accumulate some experience in remote learning which may facilitate the learning process and increase learning outcome as suggested by Orlov and colleagues (Orlov et al., 2021). To find this out, in the future, it would be advisable to continue similar research, as well as include standardized measurements of distress related to the COVID-19 pandemic. Careful examination of pitfalls of rapid change in the ways of remote teaching and learning at the universities, especially concerning students with special needs (as students with dyslexia), may help in establishing new effective teaching methods (new e-learning approach) once the COVID-19 pandemic is over.

We can expect that after the COVID-19 pandemic, hybrid/blended learning becomes a norm in the academic world. This change from traditional teaching to a new e-learning approach (understood as much broader use of different technologies than offered by remote learning) raises challenges to lecturers and students due to its magnitude and time-frame of implementation (Almaiah et al., 2020; Pham & Ho, 2020). Therefore, we should pinpoint possible advantages and disadvantages that might be faced by faculty and students, especially for those who are the most vulnerable, students with learning difficulties. We are convinced that future research needs to adopt a very broad approach and take advantage of new technologies which have been already demonstrated as useful for supporting students with learning difficulties (Pang & Jen, 2018; Perelmutter et al., 2017; Taylor et al., 2007).

## Data Availability

Data and material are available on reasonable request.

## Code availability

Code is available on reasonable request.
